# Unpacking Healthcare Utilization for Older Adults With Diabetes and ADRD in the US-Mexico Border Region

**DOI:** 10.1093/geroni/igaf122.603

**Published:** 2025-12-31

**Authors:** Alfonso Rojas-Alvarez, Hyeran Chung, Brian Downer

**Affiliations:** The University of Texas at El Paso, El Paso, Texas, United States; The University of Texas at El Paso, El Paso, Texas, United States; UTMB, Galveston, Texas, United States

## Abstract

Hispanic older adults living near the U.S.-Mexico border encounter considerable healthcare challenges that impact the management of diabetes and Alzheimer’s Disease or Related Dementias (ADRD). Studies have found that Hispanic older adults living on the U.S.-Mexico border are disproportionately affected by financial strain, limited healthcare access, and a higher prevalence of chronic diseases. Individual diagnoses of either diabetes or ADRD already present significant health challenges for Hispanic older adults, and dual diagnoses exacerbates these challenges, increasing the complexity of medical management. Using the Medicare-linked HEPESE survey (n = 718) we conduct statistical analyses to explore the relationship between dual diagnoses of ADRD and diabetes, with self-reported health measures and healthcare utilization. Our key stratifying variable is proximity to the border, given our previous findings that it explains caregiver and community resources that affect outcomes for Hispanic older adults aged 80+. Preliminary results indicate that Hispanic older adults with dual diagnoses near the US-Mexico border exhibit worse measures of healthcare utilization compared to those with a single diagnosis. Similarly, we find that Hispanic older adults with dual diagnoses near the US-Mexico border exhibit worse measures of self-reported health related to their conditions, compared to those with a single diagnosis. Structural and policy-driven factors, particularly healthcare access, influence healthcare utilization and health outcomes, with proximity to the border affecting access to care. This project is significant as it highlights the unique challenges faced by older adults with dual diagnoses in border region, aiming to inform policies that address healthcare issues for vulnerable populations.

